# Improved electrical properties of micro light-emitting diode displays by ion implantation technology

**DOI:** 10.1186/s11671-023-03819-3

**Published:** 2023-03-20

**Authors:** Yu-Hsuan Hsu, Chi-Han Wang, Xin-Dai Lin, Yi-Hsin Lin, Dong-Sing Wuu, Ray-Hua Horng

**Affiliations:** 1 Department of Photonics, College of Electrical and Computer Engineering, National Yang Ming Chiao Tung University, Xinzhu, 30010 Taiwan, ROC; 2 Institute of Electronics, College of Electrical and Computer Engineering, National Yang Ming Chiao Tung University, Xinzhu, 30010 Taiwan, ROC; 3 Department of Applied Materials and Optoelectronic Engineering, National Chi Nan University, Nantou, 54561 Taiwan, ROC

**Keywords:** Micro LED, Ion implantation, Isolation, Non-radiative recombination

## Abstract

Generally, the inductively coupled plasma-reactive ion etching (ICP-RIE) mesa technology was used to remove p-GaN/MQWs and expose n-GaN for electrical contact in a fabricated micro light-emitting diode (μLED). In this process, the exposed sidewalls were significantly damaged which result in small-sized μLED presenting a strong size-dependent influence. Lower emission intensity was observed in the μLED chip, which can be attributed to the effect of sidewall defect during etch processing. To reduce the non-radiative recombination, the ion implantation using an As^+^ source to substitute the ICP-RIE mesa process was introduced in this study. The ion implantation technology was used to isolate each chip to achieve the mesa process in the μLED fabrication. Finally, the As^+^ implant energy was optimized at 40 keV, which exhibited excellent current–voltage characteristics, including low forward voltage (3.2 V @1 mA) and low leakage current (10^–9^ A@− 5 V) of InGaN blue μLEDs. The gradual multi-energy implantation process from 10 to 40 keV can further improve the electrical properties (3.1 V @1 mA) of μLEDs, and the leakage current was also maintained at 10^–9^ A@− 5 V.

## Introduction

With the continuous development in the field of display technology, the nitride-based μ light-emitting diodes (μLEDs) have been evaluated for wide applications, such as the low-power consume wearable devices [1], wide-viewing angle outdoor displaying and televisions [2, 3], high-speed wireless visible light communication [4, 5], and high-resolution augmented/virtual reality (AR/VR) products [6]. For high pixels per inch (PPI) display, the size of μLED pixel size needs to shrink to below 20 μm. In the past years, many researchers have studied the size effect of μLEDs [7–9]. The size of the μLEDs plays a significant role in the μLED characteristics. Studies indicate that more defects related non-radiative recombination on mesa sidewalls could be observed because the defects were created during the dry etching process, especially for the μLEDs. In general, inductively coupled plasma-reactive ion etching (ICP-RIE) technology is always used to remove p-GaN/multi-quantum wells (MQWs) and expose n-GaN which is used for n-type electrical contact in μLED fabrication. In this process, much damage was induced on the exposure surface due to the bombardment from plasma-assisted dry etching. This problem of small-sized μLED shows a strong size-dependent efficiency [7–13]. Low emission intensity was observed in the μLED chips due to the effect of sidewall defects during etch processing. Meanwhile, a novel technique, atomic layer deposition (ALD), was introduced in the past decade. The passivation layer by the ALD process has higher smoothness and good step coverage. Many reports presented and confirmed that ALD can repair and passivated the sidewall defect, further reducing the non-radiative recombination and improving the electric-optical efficiency [14–16]. Recently, mesa isolation by the ion implantation process [17–19] was applied to GaN/AlGaN high electron mobility transistors (HEMTs). The mechanism is realized by the lattice disorder after ion implants, in which defects are introduced at deep levels, and decreases the electrical conductivity [20], thus accomplishing a high resistance region in GaN-based epilayers. As mentioned above, it is necessary to avoid this sidewall damage as the small size μLEDs are fabricated. In 2021, Zhe Zhuang et al. [21] also fabricated ultra-small green light μLEDs using H_2_ plasma treatment to isolate the selected p-GaN area and reduce non-radiative recombination. Jinjoo Park [13] fabricated the μLEDs using the implantation process to isolate the GaN epilayer. They demonstrated blue light μLEDs with several ion sources, such as N, Ar, Kr, Xe, and As of different implantation energies and dosages. They also indicated that ion implantation technology could confine non-radiative regions to produce relatively invariant luminance in μLEDs display. However, they only provided emission images to compare with different implantation parameters [13]. Moreover, they did not discuss the electrical properties of the ion implanted μLEDs. Therefore, the electrical properties of μLEDs by As^+^ implantation with different energy instead of mesa processing were studied to reduce the non-radiative recombination in this work. The effect of the implantation parameters on the electrical properties of μLEDs was studied. The microstructure and crystalline characteristics of μLEDs with and without ion implantation were also compared.

## Experimental

Green and blue light μLEDs were fabricated with chip sizes of 100 μm × 100 μm and 50 μm × 50 μm, respectively. Both InGaN/GaN-based LED epilayer structures were grown on (0001) sapphire substrates by the metalorganic chemical vapor deposition. There were the p-type GaN with 350 nm thickness, multi-quantum wells (MQWs) with 350 nm thickness and n-type GaN with 4 μm thickness. To obtain the *p*-type transparent Ohmic contact, a 300 nm indium tin oxide (ITO) was deposited on the p-GaN layer and then processed the thermal annealing. First, the partial ITO was etched by HCl solution to remove the ITO region where will be implanted region to replace the mesa etching. Nevertheless, the ICP-RIE was still used to etch the epilayer to expose n-GaN layer located the outside for n-metal deposition. The etching gas was Cl_2_, SiCl_4_, and Ar, and the etching rate was approximately 0.15 μm/min. Both the p-type and n-type metals were Ti/Al (50 nm/500 nm) metal layers deposited by e-gun evaporation system for the electrode contacts. After the metal deposition, the ion implantation process was introduced to the partial GaN device regions without ITO layer, which let these implanted regions between pixels become isolated. As listed in Table 1, the heavier ions of As^+^ with dosage of 1 × 10^14^/cm^2^ were carried out with a 7° tilt-angle using ion implanter at room temperature. The implantation energy will be optimized from 10 to 100 keV. The un-implanted regions with ITO layer were protected by a photoresist.

**Table 1:** Implant parameters in this study

Implanted ion	Implant energy (keV)	Dose (#/cm^2^)
As^+^	10	1 × 10^14^
	20	
	40	
	50	
	100	
	10 → 20 → 30 → 40	

The depth of ion implantation with different energies was simulated by the software of stopping and range of ions in matter (SRIM) using the GaN as target layer with a density of 6.15 g/cm^3^. Moreover, the depth of implanted samples was also analyzed by time-of-flight secondary ion mass spectra (TOF-SIMS, Germany ION-TOF, TOF-SIMS V) to compare with the simulation results. The microstructure was investigated by a field emission transmission electron microscope (FE-TEM, FEI Talos F200×) operated at voltage of 200 kV. The TEM sample was prepared using a focus ion beam (FIB, FEI Helios) technique with a Xe ion milling source. The electrical characteristics of the μLEDs were measured using the multi-function power meter KEITHLEY 2400 and micro-probes systems. The turn-on voltage, forward voltage, and leakage current at reverse voltage can be obtained from the electrical measurement. Furthermore, the dynamic resistance (@ forward voltage at 3 V) and the slope in the forward voltage region can be obtained from the current as a function of voltage (I–V) curve.

## Result and discussion

**Fig. 1:**
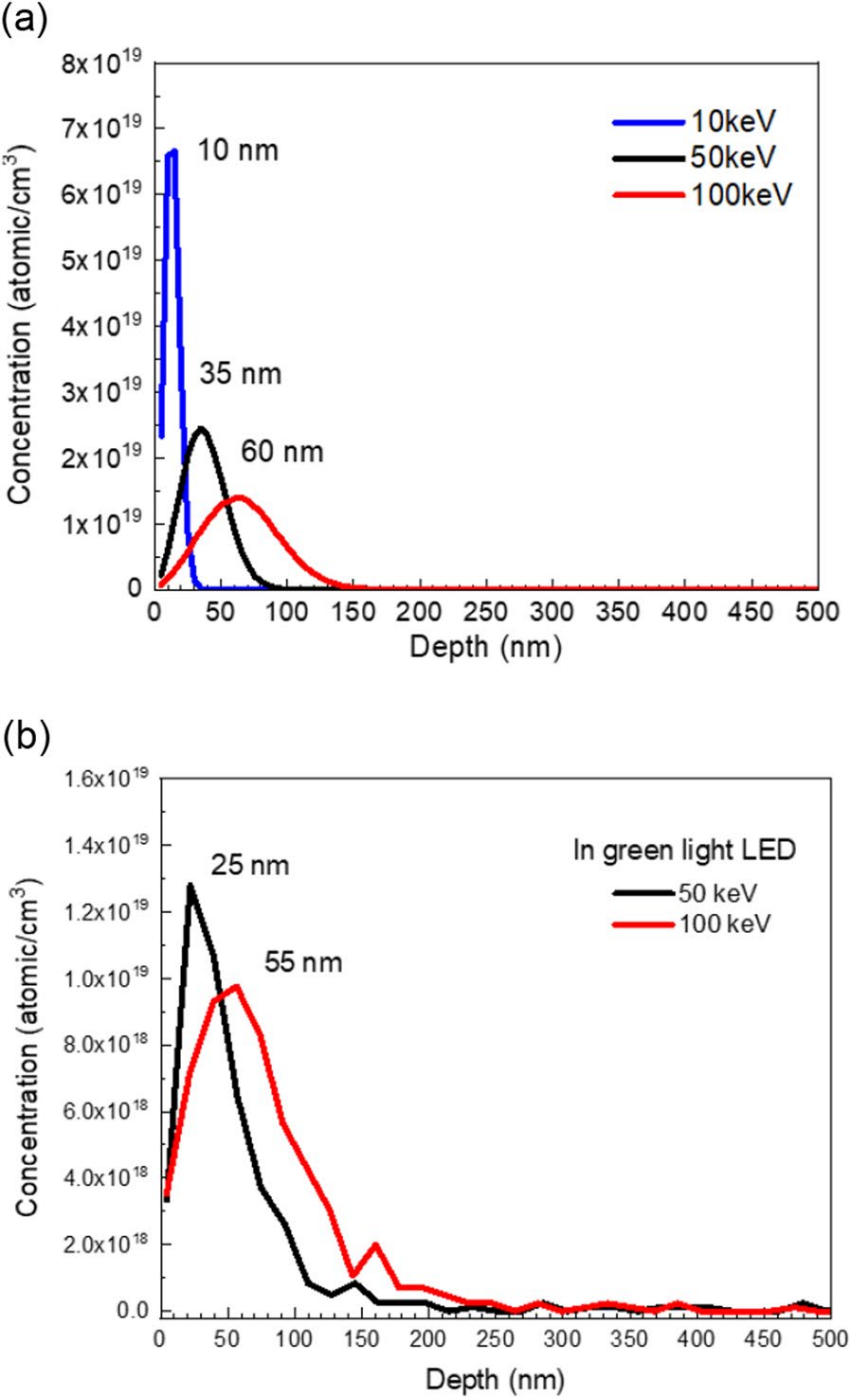
Implantation depth of **a** GaN using SRIM simulation and **b** light-emitting diode (LED) epilayer using SIMS analysis

Before manufacturing the μLEDs, the depth of different implantation energy was simulated using SRIM software, as shown in Fig. 1a. When the implantation energy increased from 10, 50 to 100 keV, the depth of the highest intensity distribution was at 10, 35, and 60 nm, respectively. A broad distribution of implant ions can be observed in high-energy parameters. Despite this, the depth was far away from μLED mesa depth. In order to evaluate the real ion implanted depth, green light LED epilayers were implanted by As^+^ with 50 and 100 keV. The corresponding SIMS measurement is shown in Fig. 1b. The highest concentration was detected at 25 nm (with 1.3 × 10^19^ cm^−3^) and 55 nm (with 1.0 × 10^19^ cm^−3^) when the implantation energy was 50 and 100 keV, respectively. It was also found that the higher implantation energy resulted in much wider area and almost stop at 230 nm below the surface. These SIMS data were consistent with the simulation results. Nevertheless, there still existed a little difference between the simulation and actual SIMS results. It could result from the difference between the simulation using the perfect crystallinity and real LEDs epilayer quality with dislocations. Obviously, the crystal with dislocations could reduce the implanted ion energy which resulted in the peak concentration depth was shallow than that of simulation. Noted that the implanted stop depth was about 160 nm and 230 nm for the As^+^ with 50 keV and 100 keV, respectively, which were less than the mesa depth for the μLED fabrication. The typical mesa depth was at least 0.7 μm.

**Fig. 2:**
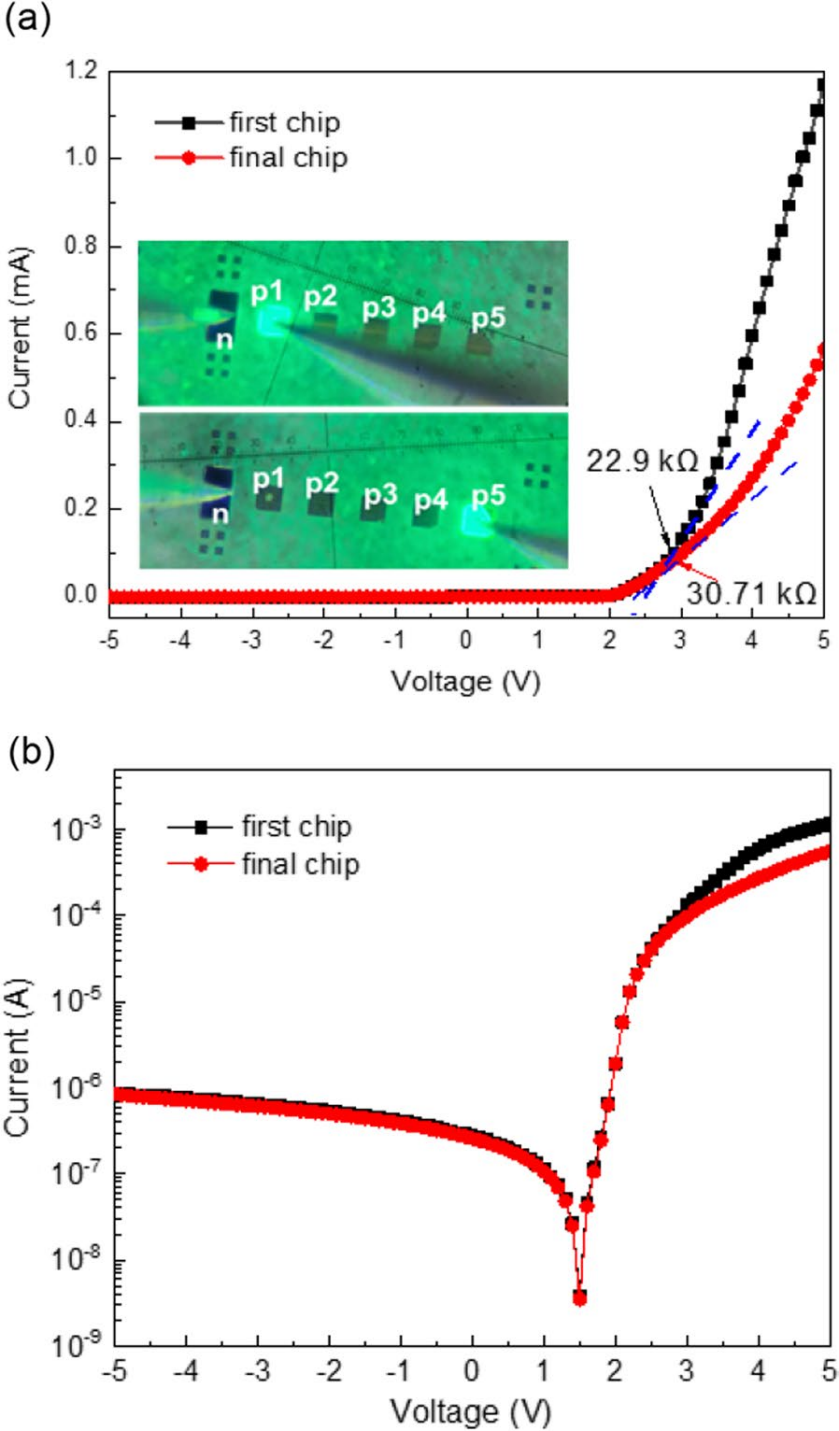
I–V characteristic in **a** linear scale and **b** log scale from − 5 to 5 V using 50 keV implantation energy

Although the depth of implantation was less than the mesa depth, it is important to evaluate the effect of ion implantation on the electrical properties of μLED. The current–voltage (I–V) characteristic of 1 × 5 μLEDs array with dimensions 100 μm × 100 μm implanted by 50 keV was measured and is shown in Fig. 2a. There were five anodes and a common cathode marked p1–p5 and n, respectively, shown in the inset of Fig. 2a for the 1 × 5 μLEDs array. Due to the different distances between the anodes and the common cathode, the forward voltages (@0.1 mA) in the first (probed at p1 and n) and the last chip (probed at p5 and n) were 2.9 and 3.1 V, respectively. At the voltage of 3 V, the corresponding dynamic resistance was 22.9 kΩ and 30.71 kΩ for the first and last μLEDs. The emission pictures are also shown in the inset of Fig. 2a. The reverse I–V characteristic is shown in Fig. 2b by log scale to present the leakage current. The leakage currents at − 5 V were very low, which was only 10^–6^ A. The above results indicated that the As^+^ ions not only damaged the p-GaN and MQW regions, but also n-GaN layers. Although the SIMS data presented the stop depth being 160 nm (about the concentration 8 × 10^17^/cm^3^, shown in Fig. 1b), the obtained IV data indicated the damaged range has exceeded the mesa depth in the green light μLED by As^+^ implantation with 50 keV energy. Furthermore, the energy of 100 keV is expected to be too deep in the mesa process. The damaged area clearly had over mesa depth, and ions destroyed the n-GaN which resulted in the decrease and isolation of n-GaN conductivity. In order to demonstrate the above inference, the n-GaN resistances with implanted energy 50 and 100 keV were also measured by the pad-to-pad method and are shown in Table 2. Before the implantation of n-GaN, the pad-to-pad resistance of n-GaN was about 2.5 kΩ.The pad-to-pad resistances of n-GaN implanted by 50 keV and 100 keV were increased to 0.03 MΩ and 0.22 MΩ, respectively. The n-GaN implanted by 100 keV was almost an order of magnitude higher than that of the n-GaN epilayer implanted by 50 keV. This result indicated that the μLED implanted As^+^ with 100 keV was too high for the mesa process.

**Table 2:** Pad-to-pad resistance of n-GaN and p-GaN (averaged by 10 chips)

Implant energy	n-GaN resistance	Implanted energy	p-GaN resistance
Before implantation	2.5 kΩ	Before implantation	15 kΩ
50 keV	0.03 MΩ	10 keV	0.04 MΩ
100 keV	0.22 MΩ	50 keV	5.06 MΩ

**Fig. 3:**
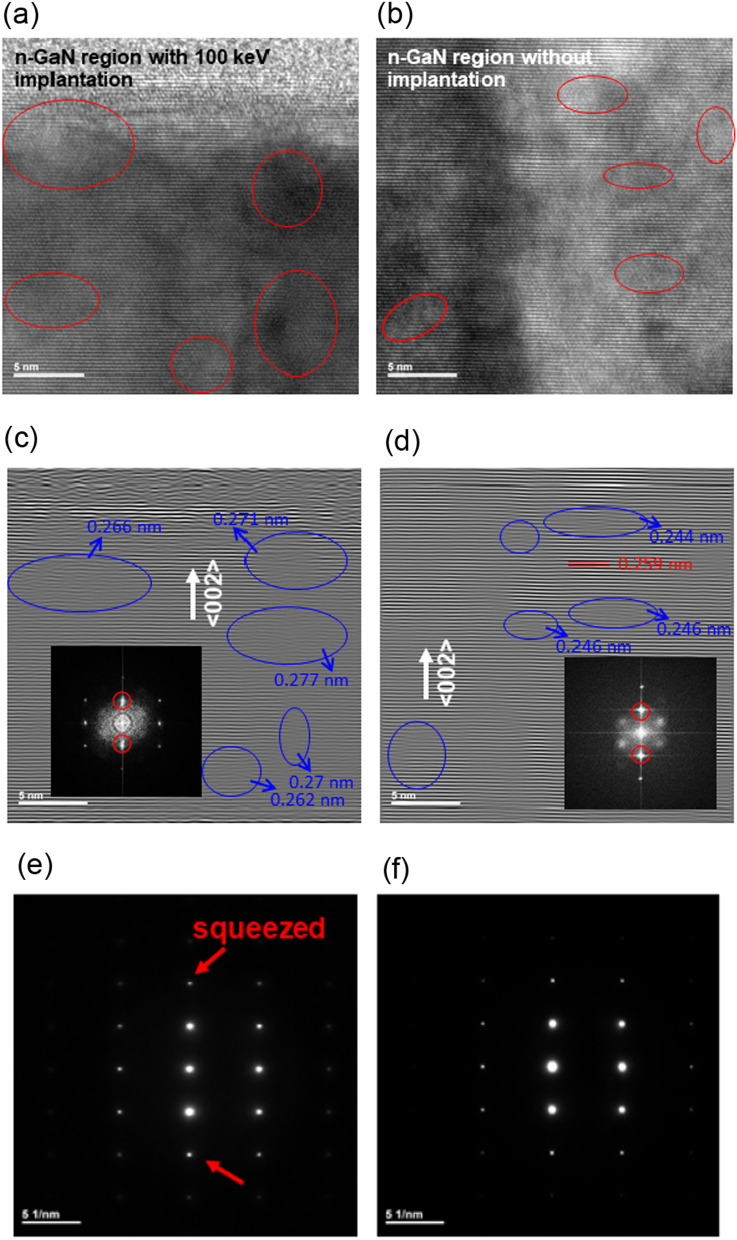
**a** Cross section high-resolution transmission electron microscopy image of 100 keV implantation, **b** without implantation, **c** IFFT of the implanted region, **d** without implantation, **e** FFT diffraction pattern of the implanted region and **f** without implantation

The microstructures of n-GaN regions with and without 100 keV implantation are shown in Fig. 3. There were some defects with the red circle marks shown in Fig. 3a and b. The density of defects was also shown in the inverse fast Fourier transform (IFFT) diffraction patterns of GaN (002) in Fig. 3c and d. After implantation, more defects and dislocations were observed and marked with blue circles, as shown in Fig. 3c. The *d*-spacing was calculated and marked beside the circles as shown in Fig. 3c and d. The *d*-spacing variated from 0.262 nm to 0.277 nm for the n-GaN implanted by As^+^ with 100 keV. In contrast, the d-spacing variated from 0.246 nm to 0.248 nm for of n-GaN without implantation. As compared with the standard GaN (002) with 0.259 nm d-spacing, the lattice distances become larger after As^+^ ion implantation due to the stretch of the crystal with the interstitial defect in Fig. 3c. However, in Fig. 3d, there were some d-spacing of defects small than standard GaN (002), which might be due to the growth stress occurring during the epitaxial process. The diffraction patterns in Fig. 3e and f show that the ion implantation slightly affects the crystallization properties using an As^+^ source with 100 keV implantation energy. Figure 3e exhibits the non-clear, squeezed patterns, which are marked with arrows. Because the crystal has been destroyed by the implanted As^+^ ions, the crystal did not symmetry and showed the “squeezed” spot in the diffraction. This indicated that the defects in the implanted sample were also induced due to using the high implant energy of 100 keV with large As^+^ ion. The obtained result was different from that presented in the previously publication [20]. In their case, the F^+^ ion was used with high implantation energy and dosage of 1 MeV and 1 × 10^15^/cm^2^; the mass of F^+^ in their study was too small; therefore, diffraction patterns did not show any difference after implantation [20]. In our study, the heavy As^+^ ion was used, which affected the diffraction pattern and slightly damaged the crystallization after the implantation, which are shown in Fig. 3e and f. Therefore, for the mesa process in μLED fabrication, the implantation energy is optimized by the electrical measurement results.

**Fig. 4:**
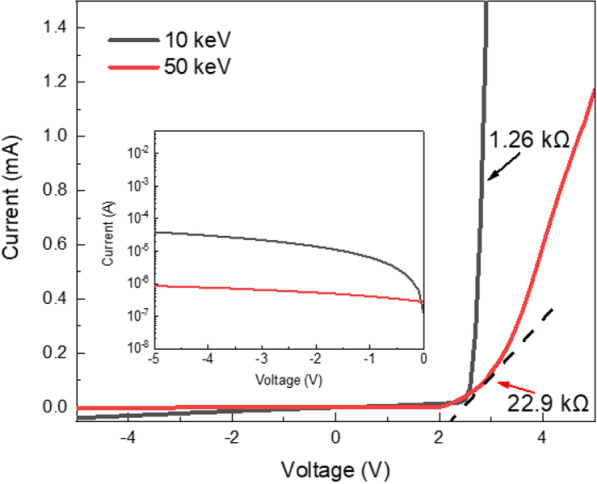
Current–voltage (I–V) curve of the green micro light-emitting diode (μLED) with implanted energy of 10 keV and 50 keV. The inset showed the log curve of the reverse current as function of reverse voltage

As mentioned above, the implantation energy of 100 keV was too high and seriously damaged n-GaN. However, 50 keV exhibits a lower leakage current, shown in the inset of Fig. 4, but obtains a slightly high n-GaN resistance and turn-on voltage. Therefore, the low ion implantation energy was further used. Figure 4 shows the I–V curve of green μLEDs implanted by 10 and 50 keV implantation energy. The dynamic resistance at 3 V voltage of green μLEDs array implanted by 10 keV energy was about 1.26 kΩ and obviously lower than 22.9 kΩ of μLEDs array implanted by 50 keV energy. Although the conductive damage resulting from the 50 keV implantation energy was lower than that due to the 100 keV implantation energy discussed in the above parts, the dynamic resistance of green μLEDs implanted by 10 keV energy was the lowest. When the injection current was 0.1 mA, the forward voltage was 2.7 V and 3.0 V at 10 keV and 50 keV, respectively. It was attributed to less damage for n-GaN implanted in 10 keV than that in 50 keV. Nevertheless, the leakage current (at − 5 V) of μLED implanted in 10 keV was about 10^–4^ A and higher than 10^–6^ A for μLED implanted in 50 keV. The p-GaN resistances were also measured using a pad-to-pad method and are listed in Table 2. Before the implantation of p-GaN, the pad-to-pad resistance of p-GaN was about 15 kΩ. The p-GaN resistance was only increased to 0.04 MΩ for μLED implanted in 10 keV, indicating that the 10 keV implantation energy was not enough to isolate the epilayer due to the shallow implant depth. Correspondingly, the p-GaN resistance was obviously increased to 5.06 MΩ for μLED implanted in 50 keV. It was also presented a little bit higher voltage due to the deeper implant depth. Based on the above results, the suitable implantation energy for the μLED mesa processing should be between 10 and 50 keV in green light epilayers using As^+^ with 1 × 10^14^/cm^2^ dosage.

**Fig. 5:**
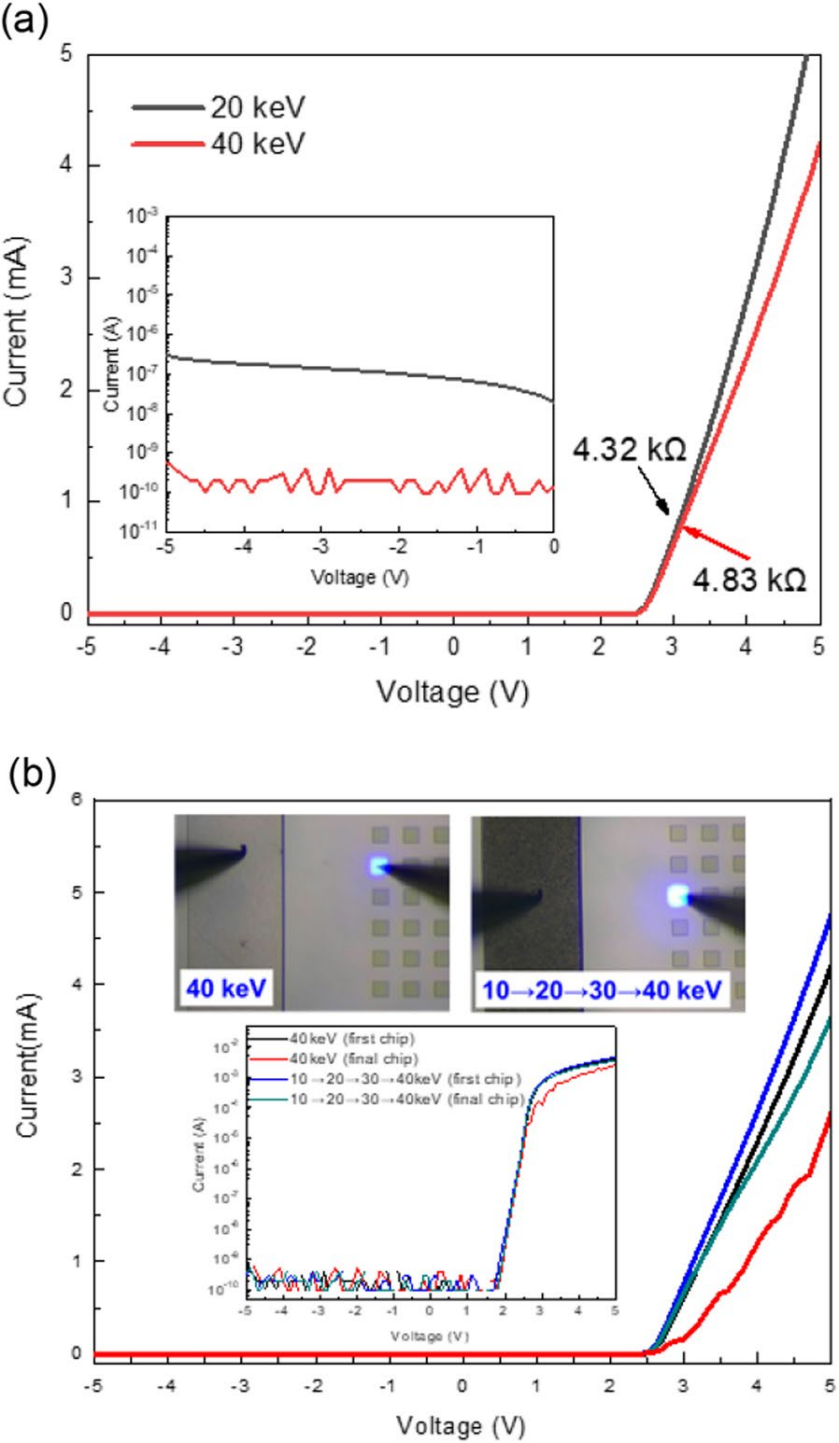
Current–voltage (I–V) curve of micro light-emitting diode (μLED) with implanted energy using **a** 20 and 40 keV, **b** multi-energies 10, 20, 30, then 40 keV. The inset of Fig. 5a shows the corresponding leakage currents. The insets of Fig. 5b show the images of turned on single chip μLEDs implanted 40 keV and multi-energies 10, 20, 30, then 40 keV currents, respectively. The I–V curve with log scale of the μLED implanted multi-energies 10, 20, 30, then 40 keV is also shown in the inset of Fig. 5b

To optimize the ion implantation energy, the implantation energy was investigated with 20 and 40 keV and then further applied to blue GaN μLEDs with 50 μm × 50 μm dimension. Figure 5a shows the I–V curve of blue μLEDs array with 96 × 48 implanted by 20 keV and 40 keV implantation energy. The forward voltage (at 1 mA injection) for the single pixel of blue μLEDs 96 × 48 array implanted by 20 keV and 40 keV was 3.1 V and 3.2 V, respectively. Nevertheless, the turn-on voltages were almost the same at 2.5 V in these two samples shown in Fig. 5a. The slope of the I–V curve at 20 keV was slightly higher than that at 40 keV, and the dynamic resistances were 4.32 kΩ and 4.83 kΩ at 3 V, respectively. The inset of Fig. 5a shows the leakage current of μLEDs implanted using these two energies. When the implantation energy was 20 and 40 keV, the leakage currents were 10^–6^ and 10^–9^ A at − 5 V, respectively. When the implantation energy was 40 keV, despite a little higher forward voltage than 20 keV, the turn-on voltage was almost identical and led to a lower leakage current. For this reason, the 40 keV was appropriate energy to mesa the μLED epilayer using an As^+^ source with a 1 × 10^14^/cm^2^ dosage.

Furthermore, the gradual multi-energy implantation method was introduced in this study and the implantation energy was increased from 10 to 40 keV, at 10 keV intervals. The gradual implantation exhibits a better electrical property with a little bit lower forward voltage in the I–V curve, as shown in Fig. 5b. Compared with single- and gradual multi-energy implantations, the forward voltages were 3.2 and 3.1 V at 1 mA current, and the dynamic resistances were 4.83 and 3.92 kΩ at 3 V, respectively. Additionally, the leakage currents were all maintained below 10^–9^ A when the reverse voltage was − 5 V. According to the inset of Fig. 5b, the emission intensity with gradual multi-energy implantations exhibited more brightness than with single implantation.

**Fig. 6:**
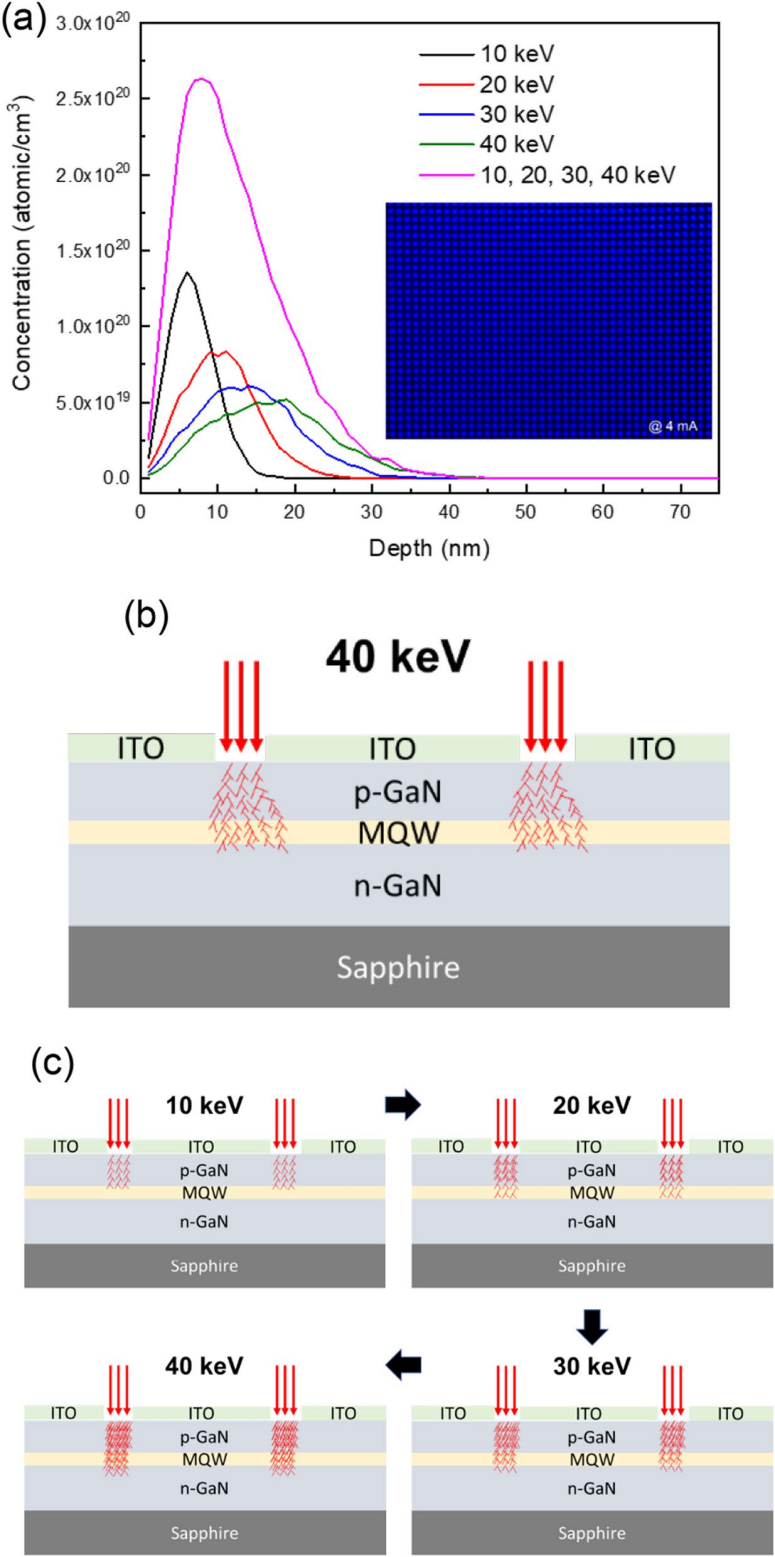
**a** Depth of implantation using stopping and range of ions in matter (SRIM) simulation of 10, 20, 30, 40 keV and multi-energy 10, 20, 30, then 40 keV and schematics of scattering effect with **b** single energy 40 keV and **c** multi-energy 10, 20, 30, then 40 keV. The inset of Fig. 6a shows the display being turned on which only emitted in the active regions

In order to optimize the ion implantation parameters, the simulated ion implantation using separate energy and multi-energy implantation are plotted in Fig. 6a. The depths were exhibited at 7, 12, 15, and 18 nm as the implantation energy increased from 10 to 40 keV. However, due to the constant dosage concentration of 1 × 10^14^/cm^2^, the area of each peak was consistent. For this reason, although the 40 keV presents a deeper implantation depth, the concentration was lower than that for a 10 keV implantation. Moreover, the low implanted energy applied to the μLEDs first could destroy the lattice. The schematics of the scattering effect are plotted in Fig. 6b and c, and as the energy increased, the high-energy ion was scattered by the destroyed lattice; consequently, it lost some energy and reduced the damage to the epilayer. Therefore, the gradual multi-energy implantation process could totally isolate the epilayer to block the current flowing and also achieve the mesa process. The inset of Fig. 6a shows the display being turned on, which only emitted in the active regions. Obviously, the gradual multi-energy implantations can play the isolation function and be instead of the mesa etching in the μLEDs fabrication.

## Conclusion

According to the SIMS analysis, the highest concentration of As^+^ was exhibited at 35 and 60 nm at 50 keV and 100 keV energies, respectively. Then, exponential decay to a deeper position occurred. The depth of the highest concentration was very shallow than the mesa depth of 0.7 μm. Nevertheless, the obtained IV characteristics indicated that the 100 keV implantation energy was too high and damaged the n-GaN layer. In contrast, 10 keV implantation energy was too low to damage the n-GaN epilayer, which was observed by pad-to-pad measurement. From IV characteristics, the forward voltage (at the rate of 0.1 mA) of μLEDs was 2.7 and 3.1 V for μLEDs implanted 10 and 50 keV energies, respectively. When the μLED was implanted by 20 and 40 keV, the two samples had a similar turn-on voltage at 2.5 V. Nevertheless, the leakage currents of the μLED implanted by 20 and 40 keV were 10^–6^ and 10^–9^ A at − 5 V, respectively.

The As^+^ implantation energy was optimized using the gradual multi-energy implantation from 10 to 40 keV with intervals of 10 keV. The simulation results showed that the concentration of multi-energy implantation was four times higher than single-energy implantation. The forward voltage improved to 3.1 V at 1 mA, and the leakage current was also maintained at 10^–9^ A at − 5 V. The obtained results indicated that the ion implantation presented a high potential to be instead of ICP-RIE for the mesa etching in the μLED fabrication process.

## Data Availability

The datasets used and/or analyzed during the current study are available from the corresponding author on reasonable request.

## Abbreviations

ICP-RIE: Inductively coupled plasma-reactive ion etching
μLEDs: Micro light-emitting diodes
AR/VR: Augmented reality/virtual reality
PPI: Pixels per inch
MQWs: Multiple quantum wells
ALD: Atomic layer deposit
HEMTs: High electron mobility transistors
ITO: Indium tin oxide
SRIM: Stopping and range of ions in matter
TOF–SIMS: Time-of-flight secondary ion mass spectra
FE-TEM: Field emission transmission electron microscope
FIB: Focus ion beam
IFFT: Inverse fast Fourier transform
